# Investigation of probabilistic optimization for tomotherapy

**DOI:** 10.1120/jacmp.v13i5.3865

**Published:** 2012-09-06

**Authors:** Michael W. Kissick, Thomas R. Mackie, Ryan T. Flynn, Xiaohu Mo, David D. Campos, Yue Yan, Donghui Zhao

**Affiliations:** ^1^ Department of Medical Physics Wisconsin Institutes for Medical Research University of Wisconsin – Madison Madison WI; ^2^ Morgridge Institute for Research Wisconsin Institutes for Discovery Madison WI; ^3^ Department of Radiation Oncology University of Iowa Hospital and Clinics Iowa City IA

**Keywords:** tumor motion, interplay, robust optimization, IMRT

## Abstract

This work builds on a suite of studies related to the ‘interplay’, or lack thereof, for respiratory motion with helical tomotherapy (HT). It helps explain why HT treatments without active motion management had clinical outcomes that matched positive expectations. An analytical calculation is performed to illuminate the frequency range for which interplay‐type dose errors could occur. Then, an experiment is performed which completes a suite of tests. The experiment shows the potential for a stable motion probability distribution function (PDF) with HT and respiratory motion. This PDF enables one to use a motion‐robust or probabilistic optimization to intrinsically include respiratory motion into the treatment planning. The reason why HT is robust to respiratory motion is related to the beam modulation sampling of the tumor motion. Because active tracking‐based motion management is more complicated for a variety of reasons, HT optimization that is robust to motion is a useful alternative for those many patients that cannot benefit from active motion management.

PACS number: 87.55.‐x, 87.56.‐v

## I. INTRODUCTION

At the University of Wisconsin Hospital Radiotherapy Clinic, many lung tumors were treated on a first‐generation helical tomotherapy (HT) machine.^(1)^ These treatments had no active motion management, and yet they were widely seen as very successful. We set out to understand why HT was more robust to respiratory motion than at first expected.

This work makes the case for further work in the direction of robust (to motion and displacement probability) treatment planning^(2)^ for HT, since it could be an acceptable alternative to active motion management techniques based on active tracking. Our argument is partly based on our previous work that demonstrates the potential for HT to exhibit only blurring motion effects on dose for a single fraction from respiration. This work adds a needed additional experimental observation, and additional theoretical reasoning and calculations, required for this argument.

The argument is as follows, with introductions to the structure of this work for each of the various components.

Given that the following have been demonstrated in our previous papers:

a) *The HT therapy beam modulation in the longitudinal (into the bore) and the transverse directions (within a slice) have dynamics that are separable because of their very disparate time scales*. This can be seen with the thread effect^(3)^ (longitudinal helical field junctioning that is robust to almost any treatment plan). It can be seen with a longitudinal Gibbs‐type deconvolution artifact that is insensitive to the transverse modulation pattern, as well.^(4)^ Motion studies with HT^5^, ^6^, ^7^, ^8^ have demonstrated the separability of response between these directions as well. b) *Our previous HT studies have included tumor motion in the following directions, and combinations of directions*: purely longitudinal,^6^, ^8^ and a combination of transverse and longitudinal.^5^, ^8^ These studies included both regular motion^5^, ^6^ and motion with randomness added,^6^, ^8^ as well as experimental motion data from patients.^(8)^ Clearly, any motion waveform can be decomposed into a series of regular sinusoidal waveforms.

The following are provided in this work to complete the picture of motion robustness for HT:

1. An analytical calculation is provided (see Methods II.B. and Results III. A.). Its purpose is to provide us with a range of motion frequencies versus gantry period frequencies to explore for an experiment. The purpose of the experiment mentioned below is to acquire the missing piece of information, as suggested by the above discussion. The analytical calculation is based on an original treatment that began the conversation of intensity‐modulated radiation therapy (IMRT) decades ago. The key aspect of the shape used for this study is that it has an equally high sensitivity to interplay effects for every direction. This symmetry is why it was introduced originally by Brahme et al.^(9)^
2. An experiment of purely transverse motion is performed for a wide range of frequencies (see Methods C., and Results B, below). By considering a wide range of frequencies, a dose error sensitivity to randomness in motion is implicitly explored.
3. A 1D illustration is provided (Methods D. and Results C.) that would demonstrate the benefits of this type of optimized inverse planning. Of course, follow‐up studies with 3D simulations and experiments on particular plans are now called for, but is beyond the scope of the current work.
4. In order to use a probability distribution function (PDF) in the treatment planning optimization, the motion was well‐sampled by the modulating beam. Such a condition is, in general, satisfied by HT. In the Discussion section (IV. A.), this analysis is presented with the help of Appendix I.
5. Finally, we discuss the big picture of this study — that we could not find any dose error interplay or interference between the transverse tumor motion and the gantry motion. The only dose errors were those caused by blurring. That means, if longitudinal interplay can be avoided, then it appears as though a single motion PDF would be applicable. Therefore, robust treatment planning based on displacement probability would seem to be efficacious for HT. This work suggests that further studies be performed to confirm that a probabilistic or motion robust planning would be a valuable alternative for patients who cannot benefit from active tracking‐based motion management. These and other practical considerations are outlined in the Discussion section.

This work ends with a call for further frequency space explorations for IMRT relative to tumor motion correction schemes and protocols. Whereas HT appears to be inherently robust to respiratory motion, more investigation is suggested by this work so that protocols based only on motion amplitude can be properly applied to HT, other arc‐based technologies, and all forms of IMRT.

## II. MATERIALS AND METHODS

### A. Tumor motion test functions

Respiration is usually dominant in the superior–inferior direction.^(10)^ The motion examined here is purely transverse to the couch motion in order to compliment previous studies with respiratory motion and HT^3^, ^5^, ^6^, ^7^, ^8^ that include both longitudinal and transverse or only longitudinal respiratory motion. In order to explore the dose error response to transverse respiratory motion over a wide range of frequencies, sinusoidal motions are explored which could represent Fourier components of any repetitive motion function with or without randomness.

The motion amplitude is dependent upon the relative gantry location. By ignoring attenuation and beam divergence, only motion perpendicular to the beam will lead to a dose variation. This same assumption is used in the first, simplest, calculation of Brahme et al.^(9)^ As a result, the simulation will not provide accurate dose error magnitudes, but it will provide the frequency ranges for which these errors will or will not be significant. In order to determine the magnitude of the dose errors, it was decided to perform experiments with the real machine. For the experiments, there is no simulated amplitude modulation, because there is a real gantry motion. A special, rotationally symmetric PTV is used for the study, which will enable a 1D approach as in this shape's original use^(9)^ if there is no interplay.

The following form was used for the analytic calculation:

(1) $$\vec{r}(t)=\vec{A}(t)\cos(\omega t+\gamma_{2}),\vec{A}(t)=[(A_{0,x}/2)\sin(\omega_{G}t+\gamma_{1}),0,0]$$

The time‐dependent breathing motion amplitude is $\vec{A}(t)$. For the experiment, the function is the following:

(2) $$\vec{x}(t)=[(A_{0,x}/2)\text{sin}(\omega t+\gamma_{2}),(A_{0,y}/2)\text{sin}(\omega t+\gamma_{2}‐\pi /2),0]$$

Where $\vec{x}(t)$ is a simple narrow ellipse (to provide some hysteresis) in the transverse plane with the long and short axes having magnitudes given as follows: $A_{0,x}=9.0mm$, $A_{0,y}=2.0mm$. The breathing angular frequency related to the breathing period is ω=2π/T, the frequency, $\omega_{G}=2\pi /T_{G}$, is the gantry rotation angular frequency related to the gantry period, $T_{G}$. There are phases $\gamma_{1}$ and $\gamma_{2}$ and both are equal to −π/4. That value gives a dose error in simulations with the high frequency limit (motion average, so zero within the PTV) equal to the low frequency limit (registration of the phantom at t=0). These values, therefore, give a condition in which the phantom is registered to the motion average.

Even though these time dependence functions are not as realistic as forms suggested by Lujan et al.,^(11)^ actual patients can be effectively simulated with a variety of functional forms.^(12)^

### B. Analytical calculation

In order to predict the general conditions in which we would expect to see interference between the collimator motion and the tumor motion (‘interplay’), we performed the following calculation. Note that IMRT and HT rely on collimator motions to modulate, but many classical theoretical papers approximate this modulation with compensator motion. The Eq. (6) and then Eq. (8) in Brahme et al.^(9)^ can be calculated with the approximation of *r* ≈ $r_{0}$ (radii close to $r_{0}$, the radius of the embedded OAR) with the relations for the dose, *D*:

(3) $$dD(r)=\frac{2}{\pi }I(x)d\varphi ,$$

(4) $$\frac{dx}{d\varphi }=z=\sqrt{x^{2}‐r^{2}}$$

**Figure 1:**
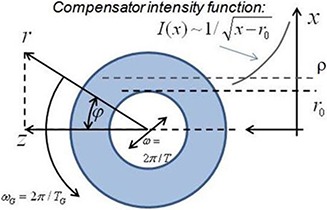
A schematic of the idealized geometry for this study, essentially identical to that presented in of Brahme et al.^(9)^ The quantity, $r_{0}$, is the distance to the edge of the central avoidance, and ρ is the radius at which the dose error is calculated, both measured from the center, and both are shown projected onto the x‐axis.

A more rigorous form is provided in the Brahme study by Eq. (18). See Fig. 1 for a pictorial description of the factors in Eqs. (3) and (4) above. Note that *I(x)* is the beam intensity shape that results from the compensator, and it has units of dose rate. The distance *x* is the position across the beam as opposed to *r* which is the distance from the center of the ring target. Since the target is registered at isocenter and to the average of the motion, it is also the distance from the isocenter. The quantity ρ is radius where the dose is calculated inside the PTV, and is greater than $r_{0}$. The gantry period is $T_{G}$, and the tumor motion period is *T*. The gantry angle is ϕ.

Using Eqs. (3) and (4), and taking the time derivative of Eq. (3) and then identifying the quantity $d\varphi /dt=2\pi /T_{G}$, yields the following for the variation of the dose:

(5) $$\Delta D=\frac{4}{T_{G}}\int_{0}^{T_{G}}I[x(t)]dt$$

With the identification of *I[x(t)]* as the dose rate of a (relatively) moving beam, and with the identification of a phase dependence, registered to the average position, this is the same relation as Eq. (9) of Bortfeld et al.^(13)^

The ring‐shaped target in Brahme et al.^(9)^ was produced with the compensator approximation (see Fig. 1), and we can therefore apply the technique outlined by Bortfeld et al. with treatment time, $T_{\text{treatment}}=T_{G}$. The dose errors from interference in this inherently nonlinear problem will then become

(6) $$\Delta D=\frac{C}{T_{G}}\int_{0}^{T_{G}}\frac{1}{\sqrt{\left(x(t)‐r_{0}\right)}}dt$$

with the approximation going relatively close to $r_{0}$ and *C* is a constant. Then, (refer to Fig. 1) using the breathing function:

(7) $$x(t)‐r_{0}=\rho +A(t)\cos(2\pi t/T+\gamma_{2})$$

By substituting Eq. (7) into Eq. (6), one gets an integral that was solved by numerical methods:

(8) $$\Delta D\propto \int_{0}^{T_{G}}{\left[\rho +A_{0}\;\text{sin}(2\pi t/T_{G}+\gamma_{1})\cdot \text{cos}(2\pi t/T+\gamma_{2})\right]}^{‐1/2}dt$$

The phases where set at $\gamma_{1}=\gamma_{2}=-\pi /4$ which correspond to a minimum average dose error at infinitely large $T_{G}/T$ that matches the static case value as $T_{G}/T\to 0$. Also (see Eq. (2)), $A_{0}=1.5cm$ and ρ=2.5cm, but it is the ratio of these values that is important. The simulations were performed with MATLAB (The MathWorks, Inc., Natick, MA).

### C. The test tomotherapy experiment

The purpose of the phantom experiment was to make sure that there are no ‘interplay’ effects over a wide range of frequency ratios between the gantry motion (15 s gantry period) and the tumor motion (breathing period spectrum components in the range 0.15 s to 150 s) in the Hi‐Art II HT machine^(14)^ (Accuray Inc., Sunnyvale, CA). The frequency ratio range explored here includes the critical range where the calculated dose error sensitivity is identified from the method described above (in Methods section B). The Washington University 4D Phantom^(15)^ was used to move a cylindrical virtual water phantom 12 cm long and 12 cm in diameter, and was moved as described in Methods section A. above.

**Figure 2:**
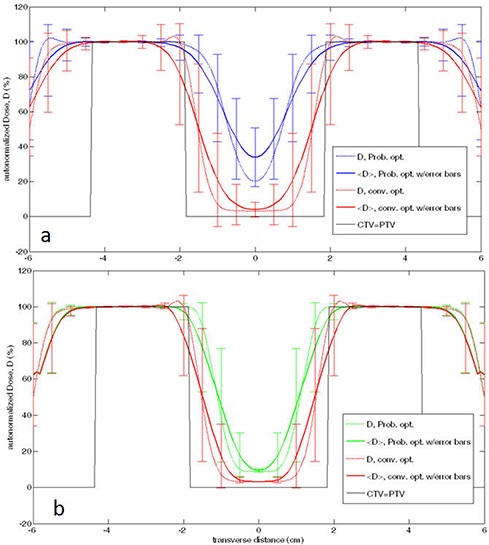
The dotted lines show the optimized dose that, when applied to the moving target, give the expectation dose (solid lines). The red lines are the conventional optimization, and the blue lines (a) are for the Gaussian motion, whereas the green lines (b) are for the sinusoidal motion. The error bars show the potential variation and decrease as the total number of fractions increases. These results are for a single fraction for which the motion PDF is constant

As Brahme et *al*.^(9)^ showed in their seminal paper, the ring‐shaped target (with a central avoidance) would provide for a strong sensitivity to motion because the beamlet strength would need to peak near the central avoidance organ‐at‐risk (OAR) structure, and this strong intensity would shoot through the PTV from every angle in the same way because of rotational symmetry. We chose this canonical shape for the experiment, as well. The outer boundary of the PTV in the transverse plane was 8.7 cm in diameter and length, and the inner boundary was 3.7 cm in diameter (see (Figs. 2a), (b), (d)). The PTV prescription was 98% of the PTV, and 60 Gy in 30 fractions. The field width was a typical 2.5 cm, and the pitch was a typical 0.287.^(3)^ The planned modulation factor (MF) was 2.0, but the actual was 1.643. The gantry period was 15.0 s. The couch speed was 0.048 cm/s.

Because it was expected that only small relative variations in dose within the PTV would be analyzed in this study (linearizable perturbations), we used old Gafchromatic EBTTM dosimetry film (International Specialty Products, Wayne, NJ). The film was technically expired and no calibration was performed to acquire absolute dose because of the uncertainties in calibrating beyond the stated expiration date. The results will show that there is no significant observed variation within the PTV, and only blurring at the boundaries of the PTV. These qualitative observations would change little with a calibration that would account for film characteristic curve that includes the inherent monotonic nonlinearity. Therefore, variations in pixel value, the raw data, were compared from one case to another, as was done in a previous paper.^(8)^ The film was scanned in the center of a large Epson 10000XL scanner in the red component. Orientations, temperature, light exposure, and handling were carefully kept consistent for all films.

### D. The proposed robust optimization scheme and 1d illustration

Following the probabilistic optimization version of robust optimization of Unkelbach and Oelfke,^(2)^ we use a quadratic objective function (see Appendix II for details) to illustrate the potential benefits one could obtain from robust (probabilistic) optimization. The rotationally symmetric PTV, the typically low pitch for tomotherapy, and the large number of beamlets with a wide range of strengths hitting each voxel^(16)^ allow for a 1D treatment if interplay or interference effects are negligible, in the spirit of the Brahme et al. study.

The expectation value of any quantity is defined by the following:

(9) $$〈D_{i}〉\equiv \sum_{j}PDF_{ij}\cdot D_{j}$$

where the ${\mathrm{PDF}}_{\text{ij}}$ is the probability of finding tissue in the voxel that was planned to be at location *i* but is actually at location *j*. In the continuous limit that is easier to work with analytically and with a spatially invariant PDF, we could write the above as:

(10) $$〈D(x)〉=\int_{‐\infty }^{\infty }PDF(x‐x\prime )D(x\prime )dx\prime$$

If we are considering the PDF to be from motion, we can identify 〈*D(x)*〉 as $D_{\text{motion}}$(*x*), the dose at position, *x*, that is convolved with motion. The PDF is created by fitting a histogram of the motion and the static dose, $D_{\text{static}}$(*x*), can be registered to any phase in the motion, such as the mean or the exhale of respiration (to name a few examples). These equations have been described before,^13^, ^15^, ^17^, ^18^ but we are suggesting here that tomotherapy, at least without dynamic jaws and dynamic couch, could be a very good technology to implement these robust optimization approaches because of its unique fluence pattern.^(16)^

In the regime where there is no 'interplay,' there will only be blurring and, therefore, the dose errors will not depend on the beamlet weights as a function of angle. The benefit of this ring shape is that the beamlet weights themselves will not depend on gantry angle. In that sense, a 1D calculation can provide a qualitative illustration of the type of benefits that could be achieved with robust (probabilistic) optimization.

**Figure 3:**
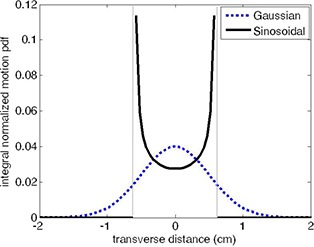
Two very different, yet realistic, PDF shapes are considered for the simulations and shown here. The sinusoidal motion PDF has a maximum motion amplitude equal to the full width half maximum (FWHM) of the Gaussian motion PDF.

In the 2D rotational case, only motions perpendicular to the transecting beams will produce dose errors (neglecting attenuation and other small effects compared to the large gradient at the lateral beam edge). In that sense, this 1D approximation will overstate the blurring. The other issue that this 1D simulation will not capture is the overlap of many beamlets such that their angles and distances from the beam center will vary considerably. To approximate this large spread in distances from the beamlet centers, a suitably large number of evenly distributed beam centers (10 used here) are used for the spacing between beamlets, meaning that they overlap by a factor of 10. The motion is simulated with a Gaussian kernel that has a 0.5 cm spread (see blue curve of Fig. 3). At another extreme in PDF shape, in order to test the sensitivity to PDF shape, a sinusoidal motion is simulated^(13)^ with the maximum extent of the motion set equal to the FWHM of the Gaussian motion PDF (see green curve of Fig. 3). Both PDF shapes are generally realistic.

The calculation is performed in MATLAB with 256 points spaced 0.5 mm apart. The beamlet size is 0.625 cm wide to approximate the lateral dimension of a tomotherapy beamlet. The motion is well‐sampled, and a single PDF is used for one fraction for which the starting phase is randomized and the expectation dose is calculated. We are therefore considering intrafractional motion‐induced dose errors in this study. For simplicity, the ring shape clinical treatment volume (CTV) is equal to the PTV, and the OAR is the central avoidance region. The robust (probabilistic) optimization scheme described above is calculated and compared to ordinary methods of optimization. Both a single dose profile and the expected dose profile for the one fraction are calculated and displayed along with the error bars of the dose profiles in Fig. 2. Increased numbers of fractions will reduce uncertainties represented by the error bars, as expected.^(13)^

## III. RESULTS

### A. Analytical calculation provides frequency ranges to explore

**Figure 4:**
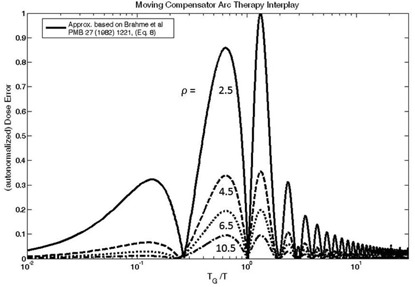
An illustration of the interference dose error response function caused by an aspect of the modulation (a single gantry rotation, in this case) having coherence with a sinusoidal tumor motion for increasing radii in the ring‐shaped PTV. The tumor motion (breathing, in this case) is assumed to remain within a plane, and so the gantry angle modulation in this case is just the projection angle variation between tumor motion and gantry motion. The phases are chosen to correspond to a registration to the long‐time average position. At large differences between these two periods, phase becomes less important to the magnitude of the dose error. In our real case with a low pitch, the gantry rotation overlaps by a factor of three. Pitch is not included in these simulations.

The results of calculating Eq. (8) with a single rotation with the parameters described in the text are displayed in Fig. 4, which shows the worst‐case scenario for interference or ‘interplay’ dose errors. This figure displays the general result that one should expect a high level of sensitivity of the dose errors from interference — maximum near $T_{G}/T\approx 1$. However, those results are for one rotation at a given initial phase. Note that the effective pitch in this calculation is unity (one gantry rotation). In reality, we typically use a pitch of 0.287 indicating about three rotations for each voxel. When one is an order of magnitude different from this condition, either higher or lower, then the dose errors from this effect are greatly reduced.

The purpose of the calculation was to find the motion frequency range of concern, and the results are that we need to perform quality assurance experiments to look for interference (or ‘interplay’) over the range $10^{-1}<T_{G}/T<10^{1}$.

### B. Experimental results

**Figure 5:**
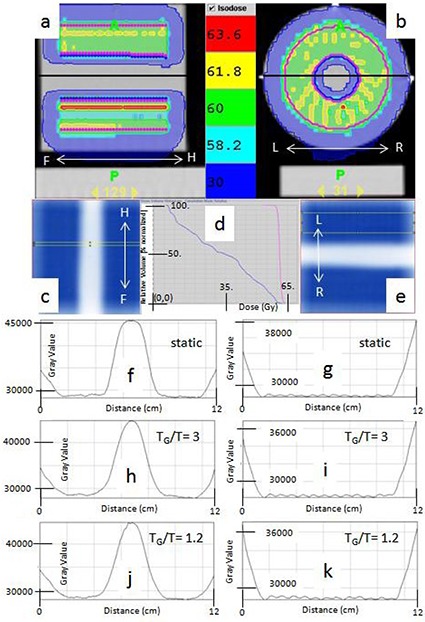
Representative preliminary results with uncalibrated Gafchromatic EBT inserted in the midplane (heavy black lines in (a) and (b)) of a HT treatment of a ring target. The plan is presented in (a), (b), and (d). The film density was analyzed with ImageJ ((c) and (e) that are from scanned film images for (a) and (b), respectively). The representative profiles shown in (c) and (e) are shown in (f)–(k). The gantry period is 15 s. The same blurring occurs for all motion frequencies tested between $0.10<T_{G}/T<10.0$.

We have performed the measurements over the range indicated above, and we see only blurring in the transverse direction (see Fig. 5). In Fig. 5, notice the yellow boxes on (c) and (e). They are used by ImageJ (National Institutes of Health, USA) to average voxels to make the profiles shown in (Figs. 5f)–(k). The profiles in (Figs. 5f), (h), and (j) are from (Fig. 5c) and all on the same plane. The profiles in (Figs. 5g), (i), and (k) are from (Fig. 5e) and all from the same plane, the midplane (heavy line) shown in (Fig. 5b). The pictures in (Figs. 5c) and (e) are scanned film images that correspond to (Figs. 5a) and (b), respectively. The important fact from these results is the lack of any noticeable interference or 'interplay,' even at frequencies where it might have occurred. For all ratios of gantry period to motion period in the range: $10^{-1}<T_{G}/T<10^{1}$. In that sense, the results here for HT for transverse motions were better for pure blurring than expected. For example, the profiles in (Figs. 5h) and (j) are basically just a blurring of the profile in (Fig. 5f). It is important to perform such a check as this over the regions of maxima shown in Fig. 4 to be sure that for any specific plan and patient, such pure blurring will be expected to hold over the power spectrum bandwidth of the patient or tumor motion.

One should note that in previous works, we concentrated on the longitudinal direction, and we and others observed interference or ‘interplay’ dose errors with the couch motion, at low breathing frequencies that are on the order of the beam width relative to the couch speed through the beam.^6^, ^8^, ^19^ That result was expected as one approaches the limit of pure blurring at a large frequency difference between modulation and tumor motion frequencies, as was mentioned in Bortfeld et al.^(13)^ with a moving compensator approximation, and also by Yang et al.^(20)^ for HT.

### C. A 1D illustration of the proposed benefits of robust optimization

One can observe in Fig. 2 that robust or probabilistic optimization will reduce the dose errors to the PTV=CTV for very different motion PDFs. The spatial dimensions are correct to the experiment, with the exception of the 2D rotation projections. These results are for a single fraction, $N_{f}=1$ (see Appendix II).

The results of this illustration (Fig. 2) show robustness to the benefits of this optimization to a variety of regular well‐sampled motions. In both the cases, the cold spots on the inside of the PTV (black lines) adjacent to the central OAR, are reduced. Note that there are no cold spots on the outsides of PTV because there is no numerical importance to the outer regions. The key issue is whether one can use a relatively constant PDF for the treatment fraction.

One can expect that the motion will blur the planned probabilistic optimization profiles better. The inner voxels of the PTV are less likely to be as underdosed (dotted blue lines in (Fig. 2a) and green lines (Fig. 2b), look near ±2cm). The current clinical situation when no motion management is used is displayed by the red lines in Fig. 2. The OAR is the central region between the PTV regions. (See Fig. 3 for the motion PDF shapes used here.) Note that a variance penalty was applied only to the PTV, and robust (probabilistic) optimization does reduce the variance in the PTV region.

## IV. DISCUSSION

### A. Helical tomotherapy beam modulation samples respiratory motion well

For HT, the dose, *D*, at a voxel at position *x* can be formulated as a beamlet‐filtered dwell time (fluence):

(11) $$D(\vec{x})=\int_{‐\infty }^{\infty }F(\vec{x}\prime )B(\vec{x}‐\vec{x}\prime )d\vec{x}\prime$$

and the *F* is the total ‘dwell time’ (units of fluence) convolved with the beamlet profile $B(\vec{x})$.

(12) $$F(\vec{x}\prime )=\int_{0}^{\infty }\psi (t)\delta (\vec{x}\prime ‐{\vec{x}}_{motion}(t))dt$$

For illustration, in Eq. (12), δ(x⇀') is a Dirac delta function that scores the crossing of the center of the beamlet, x⇀', where, like Eq. (2), ${\vec{x}}_{motion}(t)=\vec{v}t+{\vec{x}}_{tumor}(t)=\vec{v}t+\vec{A}\sin(\omega t)$ for example, with amplitude *A* and a frequency ω. The speed, $v=|\vec{v}|$, is a characteristic planned or expected speed of the device's dynamic fluence modulation. In the transverse motion case studied in this work, a dominant machine modulation speed is the gantry rotation speed: $v\sim r\cdot \omega_{G}$. In the static case with no tumor motion, ${\vec{x}}_{motion}(t)=\vec{v}t$. The units of the dwell 'time,‘ $F(\vec{x})$ are [fluence]; the units of $\delta (\vec{x})$ are 1/[x]. The units of the fluence rate, /(*t*), are [fluence]/[t]. In real life, an optimizer produces an optimized fluence rate as a function of time: a deconvolution of Eqs. (11) and (12) above with the prescription in place of *D* to deconvolve for /(*t*).

The limits of either many tumor motion cycles per beamlet or many beamlets per tumor motion cycle will allow for an approximation as follows:

(13) $$\underset{\omega ⇀\infty }{\lim}\int_{0}^{\infty }\delta (\vec{x}\prime ‐{\vec{x}}_{motion}(t))dt\approx 1/v$$

Next, let's define a motion averaged fluence rate. It still has [fluence]/[t] units, but it is only a function of space:

(14) $$〈\psi 〉(\vec{x}\prime )\equiv \int_{0}^{\infty }\psi (t)\delta (\vec{x}\prime ‐{\vec{x}}_{motion}(t))dt/\int_{0}^{\infty }\delta (\vec{x}\prime ‐{\vec{x}}_{motion}(t))dt$$

By substitution into Eqs. (11) and (12), one obtains an estimate of the blurred dose with tumor motion:

(15) $$D_{motion}(\vec{x})\approx (1/v)\int_{‐\infty }^{\infty }〈\psi 〉(\vec{x}\prime )B(\vec{x}‐\vec{x}\prime )d\vec{x}\prime$$

In other words, the dose will be a spatial integral, or a blurring, of the motion averaged fluence rate if Eq. (13) holds. It will hold if the rigid motion is well‐sampled and the PDF will be spatially invariant (see Appendix I). In such cases, robust (or probabilistic) optimization^2^, ^17^ can be a useful alternative to active tracking.^21^, ^22^

Based on this and previous works,^5^, ^6^, ^8^, ^19^ HT is inherently robust to respiratory motion interference or interplay effects. The exception, because each voxel only has one pass longitudinally through the jaws, is that low frequency longitudinal components or drifts in the longitudinal position will interfere with the couch motion.^5^, ^8^ For HT, with a typical pitch of ~1/3, and the gantry period of 15–20 sec and a breathing period of 5 sec, the value of $T_{G}/T$ for Fig. 3 is ~3–4, but even frequencies near $T_{G}/T∼1$ do not show significant interplay effects and only blurring.

### B. Implications for the clinic

The ring‐shaped target (shown in Figs. 1 and 5) used for this study represent a “worst‐case scenario” for the main issue in this paper, because of its embedded OAR that produces a concavity from all directions. It is for this reason as well that Brahme et al.^(9)^ used it on their seminal paper that started the IMRT discussion. It is difficult to achieve dose homogeneity in this case with the many large fluence gradients that must intersect the PTV at every angle. This shape has classically represented a canonical challenge for arc based IMRT like HT. Real plans will generally have concavities to a lesser degree than this shape.

#### B.1 Implications related to the application of AAPM TG‐76

It has been shown that IMRT, even very complex with simultaneous machine dynamics, in the appropriate regime where we have well‐sampled motion of an approximately constant motion PDF^5^, ^13^ results in simple “pure blurring” of the dose distribution as occurs in 3D‐conformal techniques. That is fortunate because the usual protocol, the AAPM TG‐76 report,^(10)^ suggests margins as a method for dealing with intrafraction respiratory motion, and this method implicitly assumes no interference or interplay effects. The report also suggests that we should always attempt to reduce intrafraction dose errors even though random phases together with multiple fraction will reduce dose errors over the whole treatment.^10^, ^13^

Another outcome of this work is that TG‐76^(10)^ should also include this type of frequency dependence for arc‐based IMRT in its suggested criterion to use motion management — the 5 mm motion amplitude condition.^(7)^ For example, Kim et al.^(23)^ do produce a case of leaf motion interference. Their Fig. 2 and a moving compensator approximation to IMRT could not have predicted that leaf motion interference with respiratory motion. The results by Kim et al. point to the importance of performing some form of patient‐specific quality control for IMRT, and not relying solely on margins. Other available arc therapy devices that have motion management techniques, such as Varian's RapidArc,^(24)^ can also benefit from an examination of robust optimization and a study of interference or interplay induced dose errors.

#### B.2 Implications for active motion management

It is true that robust (or probabilistic) optimization will require an invariant PDF.^(2)^ However, with more common techniques like gating with a surrogate,^(10)^ one should also ensure some predictability to the tumor motion based on a single 4D CT.^(21)^ It may be true that active tracking techniques that correct the fluence pattern to follow a known tumor motion^22^, ^25^ will provide better dosimetric corrections, but these methods can be more complicated, with the use of implanted devices and more equipment in general to coordinate during treatments, including system latency issues. Moreover, if one is using an active motion management such as tumor tracking and moving the beam or beamlets to follow the tumor, then *that itself* becomes a modulation with a rate that is coherent with the tumor motion. That is fine, as long as one can follow the phase well, because the results presented in Fig. 4 warn of the dangers of modulating at the same rate as the tumor motion. If the tracking becomes inaccurate (i.e., the phase tracking becomes inaccurate), then the dose errors could be at their maximum. Additionally, one can even use population‐based information to partially account for changes in the PDF over time^(17)^ thereby making robust (or probabilistic) optimization even more robust.

## V. CONCLUSIONS

Because active tracking based motion management may not be widely available to all patients, an alternative approach based on previous clinical success with HT, yet without motion management, is promoted. Even though this clinical success used standard HT optimization, the very same treatments would likely have also benefited from a robust or probabilistic optimization that embeds a single tumor motion PDF into the optimization process.

An argument is made in this work that this technique be considered for the HT product. This argument is based on an analytical calculation that illuminates that appropriate frequency range for concern: near $T_{G}/T∼1$. This range was explored experimentally and HT was found to be free of interplay, except for possible problems in the longitudinal direction which was explored at depth in previous papers. Because of the hundreds of beamlets striking each voxel in HT, the motion is very well‐sampled.

A warning also exists for the application of active tracking motion management. If one looses the tight correspondence of phase between the motion management scheme and the tumor motion, then one could be susceptible to larger dose errors than if one were to do nothing.

## ACKNOWLEDGMENTS

The author T. Rockwell Mackie consults for Accuray, Inc. We are grateful for the phantom provided by Scott Johnson. This work is supported by United States National Institutes of Health, National Cancer Institute grant K25 CA119344.

## APPENDICES

### Appendix I

One can derive blurring with HT from a PDF that does not vary as follows. The static dose gets blurred by the PDF if the PDF does not vary in time according to the following:

(AI.1) $$D_{motion}(x)=\int_{‐\infty }^{\infty }PDF(x‐x\prime )D_{static}(x\prime )dx\prime$$

In the static case, the time for each beam is mapped directly to *x* via the average velocity: x=vt. Therefore, the static dose is the following, and applying the commutative property of convolutions:

(AI.2) $$D_{static}(x\prime )=\int_{‐\infty }^{\infty }\psi (x\prime \prime /v_{couch})B(x\prime ‐x\prime \prime )dx\prime \prime =\int_{‐\infty }^{\infty }\psi [(x\prime ‐x\prime \prime )/v]B(x\prime \prime )dx\prime \prime$$

Substitute this into Eq. (AI.1) above, and the following results:

(AI.3) $$D_{motion}(x)=\int_{‐\infty }^{\infty }\int_{‐\infty }^{\infty }B(x\prime \prime )PDF(x‐x\prime )\psi [(x\prime ‐x\prime \prime )/v]dx\prime \prime dx\prime$$

Regroup Eq. (AI.3) as follows:

(AI.4) $$D_{motion}(x)=\int_{‐\infty }^{\infty }B(x\prime \prime )\left\{\int_{‐\infty }^{\infty }PDF(x‐x\prime )\psi [(x\prime ‐x\prime \prime )/v]dx\prime \right\}dx\prime \prime$$

Use the commutative property again to get

(AI.5) $$D_{motion}(x)=\int_{‐\infty }^{\infty }B(x\prime \prime )\left\{\int_{‐\infty }^{\infty }PDF(x\prime )\psi [(x‐x\prime ‐x\prime \prime )/v]dx\prime \right\}dx\prime \prime$$

Identify the term in brackets as

(AI.6) $$〈\psi 〉(x‐x\prime \prime )=\int_{‐\infty }^{\infty }PDF(x\prime )\psi [((x‐x\prime \prime )‐x\prime )/v]dx\prime$$

We, therefore, return this motion‐averaged fluence rate to relate to the motion dose of Eqs. (AI.4) and (AI.5):

(AI.7) $$D_{motion}(x)=\int_{‐\infty }^{\infty }〈\psi 〉[(x‐x\prime \prime )/v]B(x\prime \prime )dx\prime \prime =\int_{‐\infty }^{\infty }〈\psi 〉(x\prime \prime /v)B(x‐x\prime \prime )dx\prime \prime$$

This Eq. (AI.7) is fundamentally the same as Eq. (15).

**Appendix II**

Following Unkelbach and Oelfke,^(2)^ which describes this technique very effectively, we suggest the minimization of the following objective function, E, which is motivated by that study that optimizes a quadradic function as follows:

(AII.1) $$E=\sum_{i}\alpha_{i}^{+}C_{+}^{2}\left(〈D_{i}^{d}〉‐D_{i}^{P+}\right)+\sum_{i}\alpha_{i}^{‐}C_{‐}^{2}\left(〈D_{i}^{d}〉‐D_{i}^{P‐}\right)+\frac{1}{N_{F}}\sum_{i}\alpha_{i}^{+}\left(〈{\left(D_{i}^{d}\right)}^{2}〉‐{〈D_{i}^{d}〉}^{2}\right)$$

where the quantity $D_{i}^{P\pm }$ is the under (−) and overdose (+) penalty threshold for voxel *i*. $〈D_{i}^{d}〉$ is the expectation value of the dose delivered to voxel *i*. is the expectation value of the squared dose delivered to voxel *i*. $〈{\left(D_{i}^{d}\right)}^{2}〉$ is the under (−) and overdose (+) penalty for voxel *i*. The quantity

(AII.2) $$C_{\pm }(x)=x\cdot H(\pm x)$$

where H(x) is the Heaviside function. Finally, $N_{F}$ is the number of fractions that the patient will be treated.
